# Association between Fine Particulate Air Pollution and Daily Clinic Visits for Migraine in a Subtropical City: Taipei, Taiwan

**DOI:** 10.3390/ijerph120504697

**Published:** 2015-04-29

**Authors:** Chih-Cheng Chen, Shang-Shyue Tsai, Chun-Yuh Yang

**Affiliations:** 1 Department of Pediatrics, Kaohsiung Chang-Gung Memorial Hospital and Chang-Gung University, College of Medicine, Kaohsiung 833, Taiwan; E-Mail: charllysc@hotmail.com; 2 Department of Healthcare Administration, I-Shou University, Kaohsiung 824, Taiwan; E-Mail: sionghak@isu.edu.tw; 3 Department of Public Health, College of Health Sciences, Kaohsiung Medical University, Kaohsiung 807, Taiwan; 4 Division of Environmental Health and Occupational Medicine, National Health Research Institute, Miaoli 350, Taiwan

**Keywords:** fine particulate, air pollution, migraine, case-crossover, clinic visits

## Abstract

This study was undertaken to determine whether there was an association between fine particle (PM_2.5_) levels and daily clinic visits for migraine in Taipei, Taiwan. Daily clinic visits for migraine and ambient air pollution data for Taipei were obtained for the period from 2006–2011. The odds ratio of clinic visits was estimated using a case-crossover approach, controlling for weather variables, day of the week, seasonality, and long-term time trends. Generally, no significant associations between PM_2.5_ levels and migraine visits were observed on cool days. On warm days, however, for the single pollutant model (without adjustment for other pollutants), increased clinic visits for migraine were significantly associated with PM_2.5_ levels, with an interquartile range (IQR) rise associated with a 13% (95% CI = 8%–19%) elevation in number of migraine visits. In bi-pollutant model, PM_2.5_ remained significant after the inclusion of sulfur dioxide (SO_2_) or ozone (O_3_) on warm days. This study provides evidence that higher levels of PM_2.5_ increase the risk of clinic visits for migraine in Taipei, Taiwan.

## 1. Introduction

Over the past decade, many epidemiologic studies have demonstrated positive associations between ambient levels of airborne particulate matter (PM, generally measured as PM with an aerodynamic diameter ≤10 µm [PM_10_]) and daily mortality [1,2,3,4,5] and hospital admissions or emergency room (ER) visits for cardiovascular and respiratory morbidity [6,7,8]. The evidence on adverse effects of PM air pollution on public health has led to more stringent standards for levels of PM in outdoor air in the USA and in other countries [9].

While previous studies have primarily used PM_10_ as an exposure indicator, fine particles (defined as PM with an aerodynamic diameter less than 2.5 µm; PM_2.5_) have become a greater health and regulatory concern due to epidemiologic studies suggesting that PM_2.5_ might have greater toxicity than larger particles [10,11,12]. It is now generally accepted that PM_2.5_ are more harmful to health effect than larger particles (PM_10_) because PM_2.5_ offer a larger surface area and hence potentially larger concentrations of adsorbed or condensed toxic air pollutants per unit mass [13,14].

Relatively few epidemiologic studies (mainly focused on severe events such as mortality, hospitalizations, and ED visits) have been undertaken which address specifically the health effects of PM_2.5_, as only a few cities have monitored PM_2.5_ [15]. Very few have investigated the relationship between PM_2.5_ levels and general practitioner visits where most patients contact occur but concentrated on respiratory diseases [16,17,18,19,20]; other symptoms have rarely been investigated [21].

Migraine headache is a common clinic problem, an important cause of morbidity in mordern society [22]. Migraines represent an enormous public health concern. In United States, about 18% of women and 6% of men report migraines [23], and annual costs attributed to migtaines have been estimated to approximate $17 billion [24]. In Taiwan, a population-based survey reported that 14.4% of women and 4.5% of men reported suffering from migraine headache [25]. In addition, it was estimated that migraines account for more than 3.7 million lost working days at an estimated lost labor cost of 0.47 billion (Taiwan Dollars) yearly [26].

There are many self-reported triggers for migraines including weather, food, stress, fatigue, menstruation, and infection [27,28]. The association between air pollution or other environmental factors and migraine has not been accepted widely by clinicians [29]. A few studies have suggested that PM_2.5_ levels may be linked to migraine [30,31,32,33]. However, these results require confirmation and also further explorationas using larger datasets. 

This study was undertaken to examine the association between PM_2.5_ levels and clinic visits for migraine among people residing in Taipei city, the biggest metropolitan city of Taiwan, over the six year period from 2006–2011, using a case-crossover design. 

## 2. Materials and Methods

### 2.1. Taipei City

This study examined daily variations in clinic visits for migraine in relation to PM_2.5_ levels in Taipei for the 6-year period from 2006 through 2011. Taipei is the largest metropolitan city in Taiwan with a population of about 2.64 million and is located in northern Taiwan. The major air pollution source is automobile exhaust emission. Taipei has a subtropical climate, with an annual average temperature of 23 °C (the months with a mean temperature below 23 °C are from November through April and May through October are the months with a mean temperature above 23 °C).

### 2.2. Data Sources and Clinic Visits

The National Health Insurance (NHI) Program, which provides compulsory universal health insurance, was implemented in Taiwan on 1 March 1995. Under the NHI, 99% of the island’s population receives all forms of health care services including outpatient services, inpatient care, Chinese medicine, dental care, childbirth, physical therapy, preventive health care, home care, and rehabilitation for chronic mental illness. In cooperation with the Bureau of NHI, the National Health Research Institute (NHRI) of Taiwan has created a simple random sample of one million individuals from the entire NHI insured populations for research purposes, which cohort was further validated to be representative of the entire insured population. There were no statistically significant differences in age, gender, and healthcare costs between the sample group and all enrollees, as reported by the NHRI. This dataset (from January 1996 to December 2011) includes all claim data for these 1,000,000 subjects. These database have previously been used for epidemiological research, and information on prescription use, diagnoses, and hospitalizations has been shown to be of high quality [34,35].

With strict confidentiality guidelines being closely followed in accordance with personal electronic data protection regulations, the NHRI of Taiwan anonymized and maintained the NHI reimbursement data as files suitable for reaearch. In addition, this study was approved by the Ethics Review Board at the Kaohsiung Medical University Hospital (KMUH-IRB-exempt-20130036).

We extracted data on all clinic visits from the medical insurance file for the period 2006–2011. Cases consisted of all patients who had at least one outpatient visit with a primary diagnosis of migraine (International Classification of Diseases, 9th revision [ICD-9] code 346).

### 2.3. PM_2.5_ and Meteorological Data

Six air quality monitoring stations were established in Taipei city by the Taiwanese Environmental Protection Administration (EPA), a central governmental agency. The monitoring stations were fully automated and routinely monitor levels of five “criteria” pollutants including sulfur dioxide (SO_2_, by ultraviolet fluorescence); particulate matter (PM_10_, by beta-ray absorption); nitrogen dioxide (NO_2_, by ultraviolet fluorescence), carbon monoxide (CO, by nondispersive infrared photometry), and ozone (O_3_, by ultraviolet photometry). However, PM_2.5_ was not regularly monitored. PM_2.5_ concentrations in Taiwan have been measured continuously since 2006. PM_2.5_ was measured using tapered element oscillating microbalance method samplers. The availability of the monitoring network for PM_2.5_ provided an opportunity to investigate the impact of PM_2.5_ on clinic visits for migraine. For each day, hourly air pollution data were obtained for all of the monitoring stations. After calculating the hourly mean of each pollutant from the 6 stations, the 24-h average levels of these pollutants were computed. Daily information on mean temperature and mean humidity was provided by the Taipei Observatory of the Central Weather Bureau.

### 2.4. Statistics

The data were analyzed using the case-crossover design [36,37]. This design is an alternative to Poisson time series regression models for studying the short-term effects attributed to air pollution [38]. In general, the case-crossover design and the time-series approach yielded almost identical results [39,40,41].

We used the time-stratified approach for the case-crossover analysis [38]. A stratification of time into separate months was made to select referent days as the days falling on the same day of the week within the same month as the index day. Air pollution levels during the case period were compared with exposures occurring on all referent days. This time-stratified referent selection scheme minimizes bias due to nonstationarity of air pollution time-series data [42,43,44]. The results of previous studies indicated that the increased hospital admissions or clinic visits were associated with high air pollutant levels on the same day or the previous two days [45]. Longer lag times have rarely been described. Thus the cumulative lag up to 2 previous days (*i.e.*, the average air pollution levels of the same and previous 2 days) was used. In the analysis, the adverse health effects of each air pollutant were categorized into one of the two temperature categories: “warm” days (days with a mean temperature above the annual average temperature of the city,* i.e.*, 23 °C) and “cool” days (days with a mean temperature below the annual average temperature of the city,* i.e.*, 23 °C).

The associations between clinic visits and the levels of PM_2.5_ were estimated using the odds ratio (OR) and their 95% confidence intervals (CI) which were produced using conditional logistic regression (PROC PHREG in SAS software) with weights equal to the number of clinic visits on that day. All statistical analyses were performed using the SAS package (version 9.1; SAS Institute, Inc., Cary, NC, USA). Both single-pollutant models and multi-pollutant models were fitted with a different combination of pollutants (up to two pollutants per model) to assess the stability of the effect of PM_2.5_. Exposure levels to air pollutants were entered into the models as continuous variables. Meteorologic variables (daily average temperature and humidity on the same day) which might play a confounding role were included in the model. In all analyses, we modeled the mean temperature at lag 0 as a quadratic function, mean humidity at lag 0, and pollutants as a linear function of the 3-day moving average of current and previous 2 days concentrations (lag 0–2). Inclusion of barometric pressure did not change the effect estimates and therefore it was not considered in the final model. ORs were calculated for the interquartile difference (between the 25th and the 75th percentile) of each pollutant, as observed during the study period. To examine potential effect modification of the effect of temperature on the risk of clinic visits for migraine, we conducted analyses stratified by the cool or warm days as described above. We calculated Chi-squre statistic and corresponding two-sided p-values to assess the heterogeneity of the temperature regression coefficients from the two strata.

## 3. Results and Discussion

During the six years of the study, there were a total of 13,676 migraine clinic visits in Taipei city. The descriptive statistics for clinic visits and corresponding environmental data are shown in Table 1. The average levels of PM_2.5_ during the study period was 29.74 μg/m^3^ (there were 1226 days (56%) when the levels of PM_2.5_ were above the WHO threshold). There was an average of 6.24 daily migraine visits in the city over the study period.

**Table 1:** Distribution of daily clinic visits, weather, and air pollution variables in Taipei, Taiwan, 2006–2011.

Variable ^a^	Min	Percentile			Max	Mean
		25%	50%	75%		
PM_10_ (μg/m^3^)	14.26	34.23	45.79	61.04	888.02	50.67
PM_2.5_ (μg/m^3^)	8.35	19.28	26.93	36.76	140.54	29.74
SO_2_ (ppb)	1.00	2.58	3.51	4.72	11.14	3.79
NO_2_ (ppb)	3.22	19.86	23.65	28.35	61.94	24.44
CO (ppm)	0.13	0.49	0.62	0.78	1.99	0.66
O_3_ (ppb)	4.00	17.92	23.77	30.42	70.89	24.67
Temperature (°C)	9.05	19.33	24.07	28.49	33.18	23.60
Humidity (%)	23.56	66.68	73.13	79.70	94.19	72.86
Migraine visits	0	3	6	9	24	6.24

Abbreviation: Min, minimum value; Max, maximum value; ^a^ 24-h average.

The Pearson’s correlation coefficients among the air pollutants are presented in Table 2. Table 3 shows the effect estimates of PM_2.5_ on clinic visits for migraine in single-pollutant models and bi-pollutant models. We found evidence that the relationship between PM_2.5_ levels and migraine visits differed by season. Generally, no significant associations between PM_2.5_ levels and migraine visits were observed on cool days.

On warm days, for the single pollutant model (without adjustment for other pollutants), increased clinic visits for migraine were significantly associated with PM_2.5_ levels, with an IQR rise associated with a 13% (95% CI = 8%–19%) elevation in number of migraine visits. In bi-pollutant model, PM_2.5_ remained significant after the inclusion of SO_2_ or O_3_ on warm days.

**Table 2:** Correlation coefficients among air pollutants.

Variable	PM_10_	PM_2.5_	SO_2_	NO_2_	CO	O_3_
PM_10_	1.00	0.79	0.46	0.37	0.37	0.29
PM_2.5_	-	1.00	0.60	0.55	0.54	0.32
SO_2_	-	-	1.00	0.52	0.51	0.07
NO_2_	-	-	-	1.00	0.89	−0.06
CO	-	-	-	-	1.00	−0.22
O_3_	-	-	-	-	-	1.00

This study is one of the few that investigated the association between exposure to PM_2.5_ and clinic visits for migraine and is the first in Asia. We chose to study Taipei because it is a large city with adequate numbers of outpatient visits, and extensive air pollution data are available and our results should be applicable to other cities with similar emission sources. Data demonstrated that the levels of PM_2.5_ were positively associated with increases in the daily visits for migraine after inclusion of SO_2_ or O_3_ on warm days. The observed effects of PM_2.5_ were not maintained in the presence of NO_2_ or CO. This might be due to the collinearity between PM_2.5_ levels and concentrations of NO_2_ or CO levels, which is a common problem in this type of study.

**Table 3:** Association between PM_2.5_ exposure and clinic visits for migraine in Taipei, Taiwan, 2006–2011.

Temperature	PM_2.5_ OR (95% CI) ^a^	
≥23 °C (1222 days)	Without adjustment ^b^	1.13 (1.08–1.19)
	Adjusted for SO_2_^c^	1.15 (1.09–1.22)
	Adjusted for NO_2_^c^	1.03 (0.97–1.10)
	Adjusted for CO ^c^	1.03 (0.97–1.09)
	Adjusted for O_3_^c^	1.13 (1.07–1.20)
<23 °C (969 days)	Without adjustment ^b^	1.02 (0.98–1.07)
	Adjusted for SO_2_^c^	1.13 (1.06–1.20)
	Adjusted for NO_2_^c^	0.97 (0.92–1.02)
	Adjusted for CO ^c^	1.02 (0.96–1.08)
	Adjusted for O_3_^c^	1.03 (0.98–1.08)

^a^ Calculated for an interquartile range increase of PM_2.5_ (17.48 μg/m^3^) and adjusted for temperature and humidity; ^b^ Single pollutant model; ^c^ Bi-pollutant model.

Studies on the effect of PM_2.5_ on migraine visits are rare, and results have been inconsistent. Dales* et al.* [32] conducted a study in seven urban centers in Chile, and found no evidence of an association between migraine hospitalization and exposure to PM_2.5_. A study in Boston by Mukamal* et al.* [33] reported no significant association between PM_2.5_ and ER visits for migraine. In contrast, Szyszkowicz *et al.* [30] reported an association of 4.6% (95% CI = 1.2%–8.1%) increase in risk of daily ER migraine visits for PM_2.5_ mean level change of 8.3 μg/m^3^. A study in Edmonton, Canada, by Szyszkowicz *et al.* [31] reported an IQR (6.2 μg/m^3^) increment in the level of PM_2.5_ was associated with a 3.3% (95% CI = 0.6%–6.0%) rise in daily ER visits for migraine. In this study, a 7.44% (which corresponds to 13% increase per IQR increment) rise in daily clinic visits for migraine per 10 μg/m^3^ increment was found in the three day moving average (lag 2) concentrations of PM_2.5_ for warm days.

Because pollutants can vary considerably by season, especially O_3_ and particles, and therefore seasonal interactions between air pollutants and hospital admissions or clinic visits have often been reported. However, previous studies were conducted mostly in countries where the climates are substantially different from that in Taipei [46,47], which has a subtropical climate with no apparent 4-season cycle. Hence in this study the possible interaction of seasonality on the effects of air pollutants was not considered; but temperature was used instead. In our study, effects were observed only on warm days (effect modification). It was possible to confirm that PM effects varied by season [33]. The observed seasonal variation in effect estimates might be attributed to variation in exposure patterns. Individuals in Taipei are more likely to go outdoors and open the windows in the warm season than in cool season (higher exposure); thus, monitored PM_2.5_ concentrations may be closer to personal exposure to PM_2.5_ in the warm than cool season (better exposure assessment) [48]. This condition may attenuate the PM_2.5_-induced effects in the cool season. Furthermore, our study demonstrated greater effect estimates per unit increase of PM_2.5_ than previous reports. Variations in seasonal and regional effect estimates may in part result from differences in the chemical composition of PM_2.5_ [49]. Differences in population characteristics or PM sources, composition, absolute levels, or daily variability likely also contribute to the heterogeneity observed across studies. Of note, PM_2.5_ levels in this study were higher than those noted in previous studies. Nevertheless, the seasonal pattern of air pollution health effects need to be further investigated.

Air pollution has consistently been associated with increased hospital admissions or ER visits in cities throughout the world [50]. Recent studies suggest that the increase is due primarily to PM_2.5_ [11,51]. Major PM_2.5_ components vary by region and by season, but typically include ammonium sulfate and nitrate, elemental carbon, carbonaceous species, carbonates, metals, and water [51,52]. Despite considerable research, the relative toxicity of different constituents of PM_2.5_ remains unclear but likely varies [51].

An association between PM_2.5_ and migraine headache is biological plausible. Some pathophysiological hypotheses may be inferred to explain the association between the short-term effects of PM_2.5_ and migraine visits. PM_2.5_ have been postulated as the effective toxic fraction of PM, because they promote and maintain oxidative stress both at the respiratory level (the entry system) and at the systemic level where oxidative stress induces inflammatory reactions [53]. Cardiovascular effects may reflect neurogenic and inflammatory processes [54]. Neurogenic switching, in which exposure to irritants produces an afferent signal that triggers a distant response, potentially in a different organ system, has been hypothesized as a mechanism through which neurogenic inflammation triggered by air pollution exposures may cause migraine headache [55,56]. Alternatively, air pollutants may impair endothelial-dependent vasodialation leading to the development of migraine headaches [57]. Given the marked effects of PM_2.5_ observed in previous studies of cardiovascular disease, the role of these particles as a trigger of migraine headache clearly merits additional study [33].

The origin of chemical pollutants in an urban atmosphere is known to be principally due to road traffic [58]. PM_2.5_ concentrations have a less important natural component than PM_10_ concentrations. This smaller natural component makes PM_2.5_ a more reliable indicator than PM_10_ for measuring anthropic activity in a large city [59].

The case-crossover study design was proposed by Maclure [36] to study the effects of transient, intermittent exposures on the subsequent risk of rare acute-onset events in close temporal proximity to exposure. This design offers the ability to control many confounders by design rather than by statistical modelling. This design is an adaptation of the case-control study in which each case serves as his or her own referent. Therefore time-invariant subject-specific variables such as gender, age, underlying chronic disease, or other individual-level characteristics do not act as confounders. In addition, time-stratified approach [38] was found to be effective in controlling for seasonality, time trends, and chronic and slowly varying potential confounders [42,43,44]. In general, the case-crossover design and the general additive model (GAM) approach, which has been the analytic method of choice for studying the short-term adverse effects of air pollution since 1990 [60], produced almost identical results [39,40,41].

For a factor to confound the relationship between PM_2.5_ levels and clinic visits for migraine it needs to be correlated with both variables. It is unlikely that smoking and other indoor pollutants confound the present association since day to day variations in indoor emissions, including smoking need not be correlated with outdoor air pollution.

Exposure measurement error is a common concern in environmental epidemiology. Air pollutant levels were assigned from fixed, outdoor monitoring stations to individuals to estimate exposure. A common assumption is that the spatial distributions of certain pollutants, especially smaller particulate matter, are homogeneously distributed within large urban areas and that the concentrations between sites are well correlated. However, recent studies suggest that there may be greater variation within urban areas than previously reported [61,62]. Hence, exposure measurement errors resulting from the differences between the population-average exposure and ambient PM_2.5_ levels are not avoidable. However, the potential for misclassification of exposure due to the lack of personal measurements of fine PM exposure in this study is of the Berkson type and is known to cause a bias toward the null and an underestimate of the association [45,63]. Nonetheless, caution is advised when using central monitoring sites as proxies for population exposure in epidemiological studies without a prior analysis of spatial uniformity [61].

Our study population is homogenous in terms of race compared with populations in other cities. This study was conducted in a subtropical city. These facts may restrict somewhat the generalizability of these findings to other locations with different meteorological and racial characteristics. Furthermore, behavior such as air conditioning use or time spent outdoors may affect personal exposures. This might affect the magnitude of the observed associations in comparison with other geographic locations.

## 4. Conclusions

In summary, this study provides evidence of associations between short-term exposure to PM_2.5_ and clinic visits for migraine. The ecological design of the study precludes the inference of cause and effect. However, these findings reinforce the possible role of PM_2.5_ on clinic visits for migraine.
